# Medication Errors in an Emergency Department in a Large Teaching Hospital in Tehran

**Published:** 2013

**Authors:** Fatemeh Dabaghzadeh, Arash Rashidian, Hassan Torkamandi, Sara Alahyari, Somayaeh Hanafi, Shadi Farsaei, Mohammadreza Javadi

**Affiliations:** a *Department of Clinical Pharmacy, School of Pharmacy, Tehran University of Medical Sciences, Tehran, Iran, *; b *Department of Health Management and Economics, School of Public Health, Tehran University of Medical Sciences, Iran, *; c *Pharmaceutical Care Department, Dr. Shariati Hospital, Tehran University of Medical Sciences, Iran, *; d *School of Pharmacy, Kerman Medical University, Kerman, Iran. *; e *Research Center for Rational Use of Drugs, Tehran University of Medical Sciences, Tehran, Iran*.

## Abstract

Medication errors have important effects on increased length of hospitalization, increased mortality and costs. We assessed the incidence of medication errors and characterize the error types in an emergency department in a large teaching hospital in Tehran. We also investigated the effect of Emergency Department pharmacists on patient safety with regard to recovery of potentially harmful medication errors. The study was conducted in the 24 bed emergency

department from February to March, 2010 at a 600-bed teaching hospital. Two hospital pharmacists and two clinical pharmacy residents observed care provision and collected data on medication errors. Demographic data, type of medication error, the recorded stage of error, date and time of occurrence and report, who made the error, probability of error were recorded from medical records. We used chi-squared and independent sample t- tests for analyzing the data. We recorded 203 medication errors during 180 hours. The incidence of medication errors was 50.5% at various levels in the emergency department. Significant difference in age means was seen between the patients with and without medication errors. Seventy four point nine percent of errors were recorded as definitely an error. Most recorded errors were made by nurses (44.5%) and occurred in administrating stage (63.6%). Given that the rate of the errors was relatively high, it seems that the presence of clinical pharmacist can be beneficial.

## Introduction

A medication error is defined as a lack of success in the therapeutic process that leads to, or has the potential to lead to harm to the patient. The stages of errors include manufacturing or compounding, relevant transcribing, dispensing, prescribing and administration of a medication, and monitoring of its effects (1). Medication errors have important effects from increased length of hospitalization and costs to undue discomfort and disability or increased mortality (2). One to two percent of patients in UK and US hospitals are thought to be harmed by medication errors (3). Bates et al. reported the frequency of medication errors in 5.3% of the orders. Another study from 36 hospitals in the United States showed that 19% of administrations contained at least one error. Each error can result in an estimated cost of $5000, excluding legal expenses (4). The Emergency Department’s (ED) fast pace and unpredictability make it a high-risk area for medication errors; approximately 3% of all hospital related adverse events occur in the emergency department (5).

The large volume of patients and efforts to reduce wait times that make high pressures (5). This is not extraordinary because more than three fourth of emergency department visits are related to administrating or prescribing medications, displaying more than 210 million medication encounters annually in the United States (6). Numerous studies have demonstrated the value of pharmacists’ interventions in the emergency department (7, 8, 9). In 2007, Lada and Delgad, found that the potential cost saving associated with the interventions made by the pharmacists in ED in four months study was over $1 million (8). This study revealed that the most commonly documented interventions made by pharmacists related to the care of patients visiting the ED included recommendations about drug information, initiation of drug therapy, dosage adjustment, formulary interchanges and responses to nursing staff questions (8). Clinical pharmacists are trained in the use of medications and bring an incomparable set of knowledge and skills to the medical team (9, 10) and also can help in the selecting, obtaining, preparing, administrating, and monitoring of proper evidence-based therapeutic drug regimens (9). There are approximately 5000 emergency departments in the United States, but in less than 1% of them clinical pharmacy services are available (8). We have some studies about medication errors in Iran (4, 11-12). Fahimi and colleagues reported transcription errors in a Teaching Hospital in Iran and they concluded errors in this stage were not infrequent. (4) In another study, they evaluated the medication errors associated with infusion pumps in intensive care unit. This study was performed by pharmacists and clinical pharmacists (11). Vessal also reported prescription errors that were detected by one clinical pharmacist in a nephrology ward. Her emphasis was on the role of clinical pharmacist in the prevention of prescribing errors (12).

These mentioned studies showed the cooperation of clinical pharmacists and pharmacists with medical team were so beneficial. These studies usually have limited sample size. No study was performed in emergency department in Iran. The need for more organized study is obvious in Iran. Shariati teaching hospital is one of the first hospitals that have pharmaceutical care department in Iran. In this department both hospital pharmacists and clinical pharmacists work. This study was performed to assess the incidence of medication errors and characterize the error types in emergency department and also was to evaluate the ability of emergency department pharmacists and clinical pharmacists that supported by pharmaceutical care department on detection of medication errors.

## Experimental

*Setting*

The study was conducted in the 24 bed emergency department from February to March, 2010 at Shariati 600-bed teaching hospital affiliated to Tehran University of Medical Sciences, Tehran, Iran.

*Tools and Data collection*

The data collection team included four members (two hospital pharmacists and two clinical pharmacy residents). This team spent 6 hours per day to collect data during 30 days. Each pharmacist and each resident was responsible for 8 and 16 beds, respectively. Each member of this team recorded the patients’ data and followed them until discharge from emergency department. The members entered medication errors into paper template data forms. This form has different parts including patient data (gender, age, file number, chief complaint and etc) and type of medication error (wrong dose, wrong drug, omission errors, wrong dosage form, interactions, wrong frequency, wrong monitoring and etc). They also recorded stage of error (prescribing, administrating and monitoring), date and time of occurrence and report, who made error (physician or nurse), probability of error (probable or definite) and the name of medication. In the emergency department, the transcribing and dispensing steps are removed because of our drug delivery structure. Each member consumed 2 to 4 hours, six days in a week to review medical records and complete the forms. Each member was trained. Each team members had expertise in medications, their dosage forms, their therapeutic doses, their administering. The members recorded medication errors in regard to subjective knowledge, medical references such as uptodate and medical softwares such as lexicomp and ifact. The members of team were quite familiar with the recording process in medical records. One resident (leader) monitored the medication errors that recorded by other members. The patient’s demographics and medication information was basically obtained from medical records. One form was completed for each medication error. The definitions of each type of medication error that we used are adapted from American Society of Hospital Pharmacists guidelines on preventing medication errors in hospitals and as the followings: Prescribing errors: selecting Improper drug (based on indications, contraindications, known allergies, drug-class duplications and drugdrug interactions), dose, dosage form, quantity, route of administration, concentrations, rate of administration, or instructions for use of a drug product ordered by a physician (13-15). Wrong dose: Administration of a drug in a dose above or below the prescribed dose (13). Wrong Dosage Form: Administration of a drug in a pharmaceutical form that is different from the prescribed (13). Wrong time: administration of a dose more than 30 minutes before or after the scheduled administration time, unless there is an acceptable reason (16). Wrong Administrating technique: improper technique in the administration of a drug (14).

Unauthorized drug: Giving a non-prescribed drug (13). Wrong Frequency: When the interval prescribed by the physician was not reached to the patient correctly (4). Omission Error: The prescribed drug was not given to the patient, or the administration was not recorded (13). Wrong Route: a medication is administered to the patient using a different route than what was ordered (16). Wrong Monitoring: Failure to monitor the clinical and laboratory data before, during and after a product administration to assess the patient’s response to the prescribed medication (13). Other medication errors: Any other errors not described above.

*Data analysis*

We used Descriptive Statistics to display the percent of each type and also each level of medication errors. The Chi-Square Tests were used to assess the relationship between gender and incidence of medication errors, stage of errors and age, gender and stage of errors. Independent Samples t- test wa used to detect the difference between age means in patients with and without medication errors. The monitoring stage was removed from our analysis because of a few numbers of errors in this stage.

## Results

We observed 275 patients and recorded 203 medication errors during 180 h. Medication errors were seen in 139 medical records. Sixty two per cent and 38% of patients were male and female, respectively. The incidence of medication errors was 50.5% in the emergency department.

There was no significant difference between men and women in the incidence of medication errors. There was no relationship between gender and stage of errors. Significant difference in age means was seen between the patients with and without medication errors. The mean age of the people with and without medication errors were 58.0296 and 51.7868 respectively. The age mean in the group of patients with medication errors was 7 years more than the age mean in the group of patient without medication errors. The patients were hospitalized in emergency department for a mean of 3 days. The period between the medication error was occurred and then we reported it in most of the time (80 % of errors) was zero or one day. Between ages 50 and 75 were most likely to make errors and the most common stage of errors was prescribing in these ages. Seventy four point nine percent of errors were recorded as definitely an error. Most of errors were made by nurses (44.5%) and occurred in administrating stage (63.6%). The most common errors were omission errors (29.6%), prescribing errors (22.6%), and wrong dose (11.2%), respectively. Types of errors and their frequencies are listed in Table 1. The Detailed description of each medication error was displayed in Table 2.

**Table 1:** Type of errors and their frequency

*Stage of error – n (%)*		
67(33.0)	Prescribing	
129(63.6)	Administrating	
7(3.4)	Monitoring	
*Type of Medication Error– n (%)*		
24(11.8)	Wrong dose	
4(2.0)	Wrong Dosage Form	
4(2.0)	Duplication	
10(4.9)	Wrong Admin	
8(3.9)	Wrong Drug	
-	Known Allergy	Prescribing errors
8(3.9)	Unauthorized	
38(18.7)	Interaction	
-	Contraindication	
46(22.6)	Total	
60(29.6)	Omission Error	
9(4.4)	Wrong Frequency	
-	Wrong Route	
1(0.5)	Wrong Time	
8(3.9)	Wrong Monitoring	
39(19.2)	Other	

**Table 2:** The detailed description of each medication error

	***Age***						***Gender***		***Probability***		***Chief complaint***				***Duration A-O*** ^2^				***Duration O-R*** ^3^		
	<25	25-50		50-75		>75	M	F	P	D	Neurology	Internal	Trauma	Cardiology	0	1	2	≥3	1	2	≥3
***Stage of errors*** ^1^																					
Prescribing	1	7	37			22	38	29	51	4	15	31	1	17	37	22	4	4	15	39	10
Administrating	10	34	57			26	79	50	95	11	25	70	11	19	64	40	13	12	37	75	11
Monitoring	0	1	1			2	3	4	6	0	4	2	0	1	3	2	2	0	1	4	2
***Made by***																					
Nurse	6	29		47	22		59	47	80	9	22	57	9	16	51	34	9	12	31	65	7
Physician	3	6		33	17		36	25	46	4	17	25	2	14	31	21	5	4	9	35	13
Both	0	1		5	2		2	6	8	0	1	4	0	2	7	1	0	0	6	2	0
Patient	1	0		2	1		3	1	3	1	0	3	0	1	3	1	0	0	2	2	0
***Type of errors***																					
Wrong dose	1	8		11	4		14	10	20	1	7	12	3	2	15	6	1	2	6	13	4
Wrong Dosage Form	0	3		0	1		4	0	4	0	0	3	0	1	2	1	1	0	1	3	0
Duplication	0	0		3	1		2	2	3	1	2	2	0	0	2	2	0	0	1	3	0
Wrong Admin	0	3		5	2		5	5	10	0	2	6	1	0	8	1	1	0	3	5	1
Wrong Drug	1	0		3	4		7	1	5	1	1	6	0	1	4	3	0	1	2	4	1
Prescribing errors	0	0		0	0		0	0	0	0	0	0	0	0	0	0	0	0	0	0	0
Unauthorized Error	0	1		6	1		5	3	7	1	2	5	0	1	4	4	0	0	2	3	3
Wrong Frequency	1	2		4	2		3	6	7	0	3	4	2	0	4	3	0	2	1	8	0
Interaction errors	0	2		30	6		19	19	34	0	6	13	0	18	20	16	1	1	7	20	7
Omission errors	6	13		23	17		41	19	44	4	11	37	5	5	24	22	7	7	18	37	3
Contraindication	0	0		0	0		0	0	0	0	0	0	0	0	0	0	0	0	0	0	0
Wrong Route	0	0		0	0		0	0	0	0	0	0	0	0	0	0	0	0	0	0	0
Wrong Time	0	0		0	1		1	0	0	0	0	0	0	1	1	0	0	0	0	1	0
Wrong Monitoring	0	1		1	3		5	3	7	0	3	3	0	1	3	3	2	0	2	5	1
Other	3	11		12	11		22	17	18	7	9	18	2	8	22	9	5	3	14	19	4

## Discussion

We found a high rate of medication errors in our emergency department and observed 203 medication errors in about 180 h of observation period. Williams reported the rate of medication errors varies between 2 and 14% of patients admitted to hospital, with 1–2% of total number of patients in the US being harmed as a result (17). Marcin and colleagues reported a medication error incidence of 39% in pediatric emergency department (18). In another study (Pham *et al*) lower rate (78 reports per 100000 visits) was reported (5). Although the prescribing errors were reported the most common errors, we found that emergency department medication errors were most likely due to the wrong administration. This finding is consistent with some previous studies (5). Copp and colleagues found medication errors mostly (34%) occurred during administration inan intensive care unit (19). In one study reported the drug administration error rate of 3.5% in two medical, two surgical, and two medicines for the elderly wards in a former district general hospital (20). We found the highest portion of error related to the nurses. This result was similar to previous studies (5). Similar to Tissot and colleague (21), we inferred nurse workload can be one of the main risk factors that effect on occurrence of medication errors. We agree with Hillin that nurses must be aware of making medication errors (22). If we have future plans to educate nurses regarding medication errors and reassess their function periodically at our center, it will help us to reduce errors (23). We reported the omission errors were the most common errors in emergency department. Previous studies have shown that omission errors were one of the most common errors in different areas in hospitals such as emergency department (5, 9, 24). We found that emergency department was a crowded environment and under staffed with high workload resulted in high incidence of medication errors in emergency department. Nursing staff cannot work ideally under staff shortage and fatigue conditions. Absence of formulary interchanges at our center is apparent. Establishing such a service can reduce costs and possible errors in addition to accelerating the pharmacotherapy care. Rothschild and colleagues in their study found that emergency department’s pharmacists prevented 455 potentially harmful medication errors from reaching patients during 787 hours (6). Advance information technology (IT) in the United States make the pharmacists› intervention much easier due to better accessibility of patients› specific medical information, such as allergy profile, daily laboratory information, medical history and the most important item: medication record.

We suggest that the presence of information technology utilization in our medical centers can facilitate the positive pharmacotherapy interventions, saving lives and minimizing health care costs in long run.

A limitation of our study was that because of short observation period, we were not able to

monitor the response to therapy. Also that due the limited nature of the study, we may have missed more subjective medication errors. We concluded that hospital pharmacists and clinical pharmacists can contribute to preventing medication errors by reviewing the orders. Given that the rate of the errors was relatively high, it seems that the presence of clinical emergency department pharmacist might be cost-effective and has long term benefits such as education of nursing staff, although these needs to be assessed further in future studies. In a teaching hospital, clinical pharmacists may also contribute to the training of medical interns and residents. Previous studies have been shown the mentioned benefits of clinical pharmacists (9, 25, 26).

## References

[1] Aronson JK (2009). Medication errors: definition and classification. Br. J. Clin. Pharm.

[2] Montesi G, Lechi A (2009). Prevention of medication errors: detection and audit. Br. J. Clin. Pharm.

[3] Schachter M (2009). The epidemiology of medication errors: how many, how serious. Br. J. Clin. Pharm.

[4] Fahimi F, Abbasi Nazari M, Abrishami R, Sistanizad M, Mazidi T, Faghihi T, Soltani R, Baniasadi S (2009). Transcription errors observed in a teaching hospital. Arch. Iranian Med.

[5] Pham JK, Rodney WH, Shore AD, Shore AD, Morlock LL, Cheung DS, Kelen GD, Pronovost PJ (2011). National study on the frequency, types, causes, and consequences of voluntarily reported emergency department medication errors. J. Emer. Med.

[6] Rothschild JM, Churchill WC, Erickson A (2010). Medication errors recovered by emergency department pharmacists. Ann. Emer. Med.

[7] Vasileff HM, Whitten LE, Pink JA, Goldsworthy SJ, Angley MT (2009). The effect on medication errors of pharmacists charting medication in an emergency department. Pharmacy World and Science.

[8] Lada PA, Delgado G (2007). Documentation of pharmacists’ interventions in an emergency department and associated cost avoidance. Am. J. Health-Sys.Pharm.

[9] Christopher EJ, Karalea JD, Daniel HP (2010). Clinical pharmacists: coming soon to an emergency department near you. Adv. Emer. Nurs. J.

[10] Aronson JK (2009). Medication errors: emerging solutions. Br. J. Clin. Pharmacol.

[11] Fahimi F, Sistanizad M, Abrishami R, Baniasadi S (2007). An observational study of errors related to the preparation and administration of medications given by infusion devices in a teaching hospital. Iran. J.Pharm. Res.

[12] Vessal G (2010). Detection of prescription errors by a unitbased clinical pharmacist in a nephrology ward. Pharmacy World and Science.

[13] Cruz Belela AS, Sorgini Peterlini MA, Gonçalves Pedreira ML (2010). Disclosure of medication error in a pediatric intensive care unit. Revista Brasileira deTerapia Intensiva.

[14] American Society of Hospital Pharmacists (1993). ASHP guidelines on preventing medication errors in hospitals. Am. J. Hospital Pharm.

[15] Azoulay L, Zargarzadeh A, Salahshouri Z, Oraichi D, Bérard A (2005). Inappropriate medication prescribing in community-dwelling elderly people living in Iran. Eur.J. Clin. Pharmacol.

[16] Flynn EA (1999). A Brief History of Medication Errors. American Pharmaceutical Association.

[17] Williams DJP (2007). Medication errors. J. Royal College ofPhysicians of Edinburgh.

[18] Marcin JP, Dharmar M, Cho M, Seifert LL, Cook JL, Cole SL, Nasrollahzadeh F, Romano PS (2007). Medication errors among acutely ill and injured children treated in rural emergency departments. Ann. Emerg. Med.

[19] Kopp BJ, Erstad BL, Allen ME, Theodorou AA, Priestley G (2006). Medication errors and adverse drug events in an intensive care unit: Direct observation approach for detection. Crit. Care Med.

[20] Ridge KW, Jenkins DB, Noyce PR, Barber ND (1995). Medication errors during hospital drug rounds. Quality in Health Care.

[21] Hillin E, Hicks RW (2010). Medication errors from an emergency room setting: safety solutions for nurses. Crit. Care Nurs. Clin. North Am.

[22] Tissot E, Cornette C, Limat S, Mourand JL, Becker M, Etievent JP, Dupond JL, Jacquet M, Woronoff- Lemsi MC (2003). Observational study of potential risk factors of medication administration errors. Pharmacy World and Science.

[23] Abbasi Nazari M, Salamzadeh J, Hajebi G, Gilbert B (2011). The role of clinical pharmacists in educating nurses to reduce drug-food interactions (absorption phase) in hospitalized patients.

[24] Barker KN, Flynn EA, Pepper GA, Bates DW, Mikeal RL (2002). Medication errors observed in 36 health care facilities. Arch. Intern. Med.

[25] Kaboli PJ, Hoth AB, McClimon BJ, Schnipper JL (2006). Clinical pharmacists and inpatient medical care. Arch. Intern. Med.

[26] Fahimi F (2010). Implementation of a clinical pharmacy education program in a teaching hospital: resident oriented documentation and intervention. Iran. J. Pharm. Res.

